# The ontology of mental health disorders: embracing the diathesis-stress model

**DOI:** 10.31744/einstein_journal/2026AE1796

**Published:** 2025-12-04

**Authors:** André Connor de Méo Luiz, Myenne Mieko Ayres Tsutsumi

**Affiliations:** 1 Instituto Continuum Londrina PR Brazil Instituto Continuum, Londrina, PR, Brazil.; 2 Pontificia Universidade Católica do Paraná Londrina PR Brazil Pontificia Universidade Católica do Paraná, Londrina, PR, Brazil.

**Keywords:** Diathesis-stress model, Mental disorders, Behaviorism, Contextualism, Mental health, Stress vulnerability

## Abstract

This conceptual article examines the ontological and epistemological foundations of mental health disorders through the lens of the diathesis-stress model. It seeks to articulate how this model contributes to a more comprehensive and context-sensitive understanding of psychological suffering, moving beyond reductionist, biological explanations. Grounded in a radical behaviorist and contextualist framework, this article integrates historical, clinical, and philosophical perspectives to demonstrate the relevance of the diathesis-stress paradigm for assessment, diagnosis, intervention, and prevention in mental healthcare. The goal is to establish the model as a foundational tool for interdisciplinary collaboration and propose future directions for its use in research and applied settings. In summary, this article advocates the adoption of the diathesis-stress model as a fundamental framework for understanding the complexity of mental health disorders and its practical implications for mental health.

## Highlights

■ This paper proposes a contextual and non-reductionist ontology of mental health disorders.
■ It reframes the diathesis-stress model through radical behaviorism and functional contextualism.
■ It emphasizes dynamic interactions between vulnerabilities and environmental stressors.
■ It guides clinical practice toward integrative, personalized, and system-sensitive interventions.

## Summary

Mental health and psychological suffering arise from the dynamic interplay between interdependent biological, psychological, and environmental systems. The diathesis-stress model emphasizes that behavior emerges from continuous transactions between individuals and their contexts, which are shaped by historical vulnerabilities and evolving stressors. In this view, diagnosis aims not to label but to map the functional patterns within these interactions.

## Graphical abstract

**Figure:**
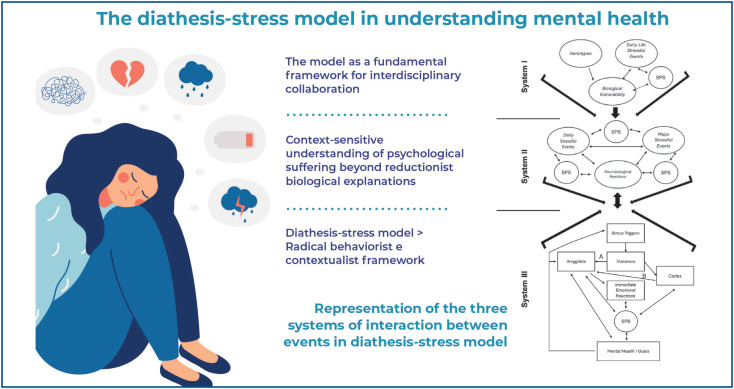

## INTRODUCTION

The conceptualization of mental health has undergone a profound transformation over the centuries. In the past, non-scientific perspectives dominated, and metaphysical or supernatural explanations, such as demonic possession or divine retribution, were the prevailing narratives.^(1-3)^ Consequently, the interventions employed were often rooted in religious or superstitious practices, rather than being grounded in empirical research.

As the scientific era progressed, the focus shifted towards the biological and physiological examination of mental health disorders. Researchers and clinicians began to investigate the concrete and organic basis of mental illnesses, such as structural abnormalities in the brain or chemical imbalances.^(4-6)^ While this approach led to significant advancements in pharmacological treatments and surgical interventions, it soon became apparent that a singular emphasis on biological determinants was insufficient.^(7,8)^ The complexity of mental health disorders necessitated an integrated perspective that could also account for psychological dispositions and social contexts. This refinement resulted from the evolution of the biopsychosocial model, which integrated the biological, psychological, and social dimensions into a unified framework. Within this paradigm, the diathesis-stress model is a particularly insightful conceptual tool, as it posits that mental disorders arise from the interplay between preexisting vulnerabilities and the environment.^(9-11)^

The diathesis-stress model represents a conceptual and practical paradigm shift in the understanding, diagnosis, and treatment of mental-health disorders.^(11-13)^ The model emerged in the field of the study of schizophrenia,^(14,15)^ and in contrast to models solely rooted in physiological determinism, which attribute the origins of mental illness to discrete biological aberrations, it posits that mental disorders arise from the dynamic interplay between predisposing vulnerabilities and environmental stressors.^(16,17)^ Thus, it is a model of psychopathology that conceptualizes the development of psychological disorders as a result of an interaction between an individual's inherent vulnerability and exposure to stressful life events.^(18)^ Additionally, this model relies on a fundamentally non-dualistic and interactionist ontology, positing that neither intrinsic traits nor external stressors alone are sufficient for the emergence of psychopathology; rather, it is the dynamic interplay between inherited predispositions and temporally situated stressors that accounts for psychological dysfunction.^(19)^ From this perspective, the diathesis-stress framework is not merely a clinical heuristic but a philosophical model that embodies a functional-contextual view of human behavior, emphasizing variability, plasticity, and historical contingency as central features of psychopathological development.^(20)^

## A multifactorial framework: the nuance of vulnerability

The term "diathesis" encompasses a wide range of predisposing factors that heighten an individual's vulnerability to developing mental health disorders.^(21)^ These predispositions may be biological, such as genetic variations in serotonin transporter genes, hormonal imbalances, or neurodevelopmental alterations; psychological, including rigid cognitive patterns, emotional dysregulation, perfectionistic tendencies, or learned helplessness; and social, comprising early attachment disruptions, experiences of abuse or neglect, exposure to chronic discrimination, or unresolved cultural identity conflicts.^(22)^ Contemporary psychological science conceptualizes diathesis not as a fixed trait or pathology but rather as a dynamic, multifactorial profile shaped by the interplay between genetic and environmental influences across an individual's developmental history.

Importantly, these vulnerabilities do not inevitably lead to psychopathology. Rather, they are probabilistic in nature, modulating the likelihood that an individual will develop psychological difficulties when encountering certain environmental demands or stressors.^(23)^ This perspective underscores the plasticity of human behavior and the possibility of resilience, even in the face of significant vulnerability. For instance, a genetic predisposition toward anxiety may remain dormant throughout an individual's life if protective environmental conditions are in place.^(16)^

From this viewpoint, diathesis is better conceptualized as a sensitivity profile, in which the degree of vulnerability varies across individuals and within the same individual over time, depending on factors such as developmental stage, hormonal shifts, learned coping strategies, and other contextual influences. For example, a person with a family history of mood disorders, poor emotional regulation, and low distress tolerance may never experience a depressive episode unless exposed to prolonged, high levels of stress such as financial instability, chronic illness, or the rupture of significant relationships.^(24)^ Conversely, another individual with fewer risk factors and a history of adaptive coping may endure similar or even more intense stressors without developing clinically significant symptoms.^(17)^

The stress component functions as a triggering mechanism, activating latent vulnerabilities, and initiating or exacerbating the symptoms of mental distress.^(25)^ However, the impact of a given stressor may vary considerably across individuals. For instance, when two people experience job loss, one may interpret it as a temporary setback and engage in problem-solving strategies, while the other may spiral into despair, particularly if predisposed to negative attributional styles or low self-efficacy. This variability underscores the central tenet of the model; individual differences in diathesis determine the threshold at which stressors become pathogenic. Furthermore, the relationship between diathesis and stress is bidirectional and reciprocal.^(26)^ Not only can stressors activate vulnerabilities, but the vulnerabilities themselves can also increase the likelihood of encountering stressors, a phenomenon known as stress generation. For example, individuals with high rejection sensitivity may inadvertently engage in behaviors that lead to social exclusion, thereby reinforcing the stress that perpetuates their condition.

**Figure 1:**
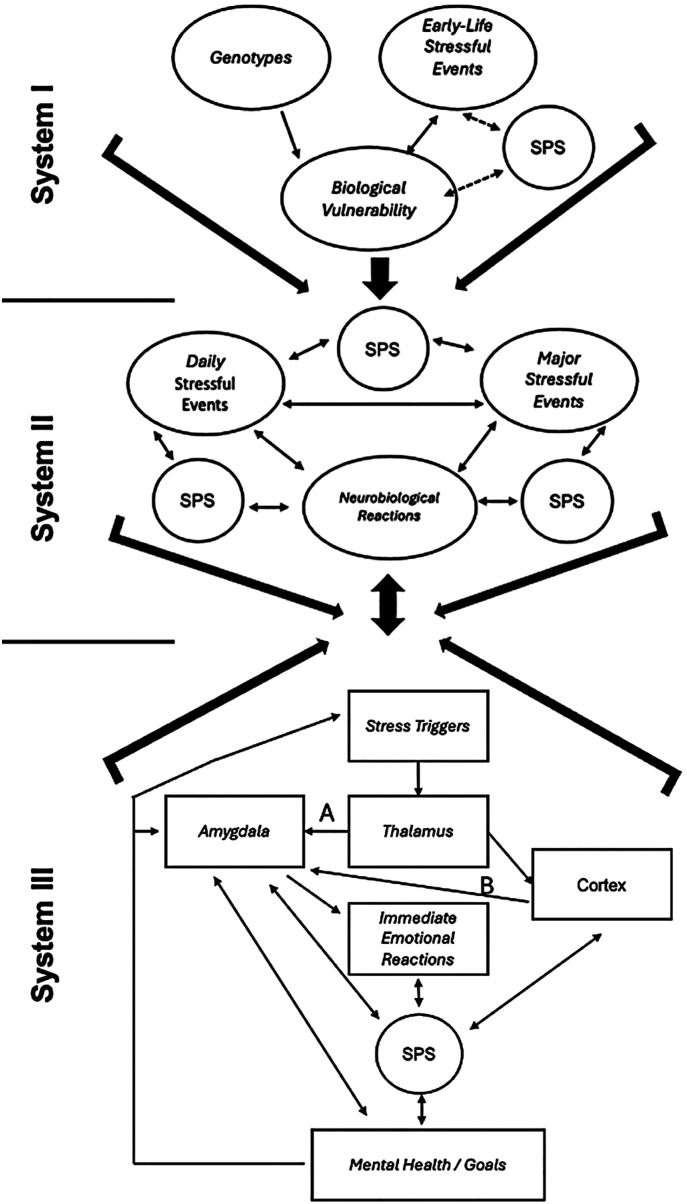
Representation of the three systems of interaction between events in the diathesis-stress model

To further refine the understanding of the diathesis-stress model and its clinical applications, it is helpful to consider the interactions between genetic factors, life experiences, problem-solving capacities, and neurobiological responses to stress. The model expands the concept of vulnerability (diathesis) by conceptualizing it as the result of multiple historical and current interactions between an organism and its environment, including public, private, and physiological elements. The diathesis-stress model comprises three interdependent systems. These systems are illustrated in figure 1. Nezu et al. developed the logic of the diagram presented in figure 1 in their treatment manual,^(11)^ but we recreated the presentation in this figure.

SPS: social problem solving.

Source: adaptad from Nezu AM, Nezu CM, D’Zurilla TJ. Problem-solving therapy: a treatment manual. Cham: Springer Publishing Company; 2013. 323 p.

System I includes genetic factors and early life stressors that directly influence the development of biology and behavior.^(27-29)^ These vulnerabilities set the stage for System II, which involves significant and daily stressors during adolescence and adulthood, as well as the neurobiological reactions evoked by such events.^(30,31)^ System III represents the micro-level analysis of immediate neurophysiological responses triggered by stressors, such as activation of the amygdala, thalamus, and cortex. These responses are understood as evolutionary processes that prepare the organism for action; however, when chronically overactivated and not accompanied by effective coping repertoires, they increase the risk of developing psychopathology.^(32-34)^ From this perspective, biological vulnerabilities and deficits in problem-solving abilities reinforce one another, exacerbating sensitivity to stress and hindering the implementation of adaptive responses.

Thus, the relationship between diathesis and stress is circular and dynamic; vulnerabilities increase the likelihood of exposure to stressors (a phenomenon known as stress generation), whereas repeated exposure to stressors without effective coping strategies amplifies neurophysiological responses and the risk of mental illness. In this context, clinical intervention must consider the manifested symptoms, the history of interaction among systems, and the coping skills that the individual has or has not developed over time. This functional and interactionist perspective of diathesis and stress reinforces the usefulness of the model as a powerful clinical guide. By mapping the systems involved in vulnerability and stress reactivity, therapists can design personalized interventions to promote more effective problem-solving and emotional regulation repertoires.

The diathesis-stress model presents a multifaceted, interactionist perspective on mental health that departs significantly from simplistic or reductive explanations. This framework prompts mental health professionals to investigate how individual predispositions and environmental factors dynamically shape emotional and behavioral patterns over time. Rather than searching for a single "cause" of mental illness, clinicians operating within this model aim to develop an individualized, context-sensitive case formulation that accounts for an individual's history and current life circumstances. This approach encourages the design of tailored, integrative interventions that extend beyond symptom management to foster long-term psychological resilience.

## Clinical utility: guiding assessment, diagnosis, and intervention

The diathesis-stress model provides a robust framework for guiding clinical decision-making by incorporating the complexity of human behavior and context into assessment, formulation, and intervention strategies. In contrast to the physiological model, which often prompts immediate pharmacological intervention based on symptom clusters and presumed neurochemical dysregulation, the diathesis-stress model encourages clinicians to adopt a formulation-based approach grounded in individualized understanding.^(9,35)^ This approach involves identifying each client's unique vulnerability profile and considering their genetic predispositions, psychological tendencies, and social and cultural influences. It also includes mapping the environmental stressors that contribute to the onset, maintenance, or exacerbation of symptoms, and designing personalized interventions that strategically reduce vulnerabilities, buffering the impact of stressors, or—ideally—addressing both simultaneously.^(36-38)^

Clinicians operating within this framework begin by conducting a comprehensive and integrative biopsychosocial assessment. Rather than relying solely on categorical diagnoses or symptom checklists, they investigate various factors, such as medical and psychiatric history, current psychological functioning, family dynamics, social networks, cultural identity, and the chronology of significant life events.^(39,40)^ While the physiological model often prioritizes rapid symptom classification and medication history, the diathesis-stress model emphasizes functionality across life domains and views behavior as an outcome of intersecting historical, contextual, and environmental variables.

Consider, for example, a client presenting with panic symptoms. A clinician operating from a strictly biological perspective might attribute this to a neurotransmitter imbalance and promptly prescribe anxiolytics. In contrast, a professional using the diathesis-stress model would explore whether the client exhibits a temperament marked by heightened neuroticism, a history of trauma or early relational disruptions, and exposure to ongoing stressors such as workplace instability or caregiving burden. The resulting treatment plan, rather than being exclusively pharmacological, would likely include psychoeducation about the stress response and physiological arousal, cognitive-behavioral therapy for cognitive reappraisal, and exposure-based work, and—when clinically indicated—pharmacological support may be used as one of the several tools in a broader, layered intervention.

The choice of treatment approach can vary significantly depending on the clinician's theoretical orientation. For example, a physiological perspective may view a depressive episode as stemming from serotonin deficiency, leading to the primary use of selective serotonin reuptake inhibitors.^(4,41)^ While such medications can be effective and necessary, relying solely on this approach may overlook the importance of contextual and psychological factors such as financial hardship, caregiver fatigue, dysfunctional beliefs, or deficits in emotion regulation. By contrast, the diathesis-stress model encourages clinicians to formulate problems and solutions across multiple domains. Interventions can encompass biological aspects, such as using medication to alleviate 1) acute symptom intensity in cases of high neurochemical sensitivity; 2) psychological aspects through evidence-based therapies such as emotion-centered problem-solving therapy^(12)^ and acceptance and commitment therapy^(42)^ to restructure maladaptive thinking and enhance resilience; and social aspects through systemic interventions such as family therapy, case management, or resource navigation to address external stressors and reinforce protective environmental factors.

Notably, the diathesis-stress model does not discard physiological insights. Instead, it integrates biological data as one component within a broader, behaviorally grounded, and contextually sensitive framework. This systems-oriented view aligns with contemporary clinical science, particularly with functionally oriented approaches that emphasize the identification of contingencies maintaining behavior and the analysis of environmental conditions contributing to psychological difficulties. Beyond assessment and intervention, the diathesis-stress model significantly expands the scope of mental healthcare through its contributions to prevention and psychoeducation. For individuals identified as high-risk, whether due to genetic predisposition, a history of developmental adversity, or exposure to structural inequalities, early, targeted interventions can be implemented to strengthen coping resources and reduce the likelihood of symptom development.^(43)^ In this context, psychoeducation can be informative and empowering, helping clients gain a better understanding of how their vulnerabilities interact with environmental pressures. This insight can help reduce self-stigma, increase treatment adherence, and promote emotional self-regulation, structured problem solving, and anticipatory coping skills.^(37)^

## Epistemological and philosophical implications

From an ontological perspective, the diathesis-stress model departs significantly from the reductionist paradigms that conceptualize mental disorders solely as "brain diseases" or deficits in neurochemical functioning.^(9,10)^ Rather than viewing psychological suffering as a direct product of faulty brain mechanisms, this model proposes that mental health and its breakdown emerge from the dynamic interplay of multiple interdependent systems. These systems encompass neurobiology, environmental contingencies, interpersonal relationships, cultural practices, historical reinforcement patterns, and an individual's learning history.^(44,45)^ This broader, systems-oriented ontology resists simplistic cause-effect explanations and instead situates behavior, including maladaptive patterns, within a richly contextual framework.

This ontological shift has deep implications for understanding the nature of mental disorders, goals of diagnosis, locus of responsibility, and standards by which treatment efficacy is evaluated. If psychological suffering arises from complex and interactive systems, then the purpose of diagnosis is not merely to categorize internal dysfunctions but also to identify patterns of interaction between the individual and their environment.^(46)^ Consequently, responsibility for recovery cannot rest solely on the individual's biology or willpower but must be distributed across multiple contexts—familial, societal, and institutional—in which the person is embedded. In this view, treatment efficacy is not solely defined by symptom suppression but by restoring or enhancing functional, flexible, and meaningful interactions with one's environment.^(47,48)^

Epistemologically, the model is compatible with a radical behaviorist framework^(49)^ particularly when conceptualized through the lens of functional contextualism.^(50)^ Radical behaviorism is a philosophical framework for understanding behavior, developed primarily by Skinner.^(51)^ It serves as the foundation of the science of behavioral analysis.^(52,53)^ Unlike other forms of behaviorism (*e.g.*, Watsonian or methodological behaviorism), radical behaviorism is distinguished by its inclusion of private events—thoughts, emotions, and sensations—as part of the behavioral stream, being different only in how each behavior is accessed:^(54,55)^ public behaviors are those directly accessed by others, and private behaviors are those directly accessed only by the person that emits the behavior. However, both private and public behaviors have the same nature and are caused by similar processes.^(56,57)^

Thus, at its core, radical behaviorism is grounded in the assumption that behavior—whether public (e.g., speaking, running) or private (*e.g.*, thinking, feeling)—is a natural phenomenon that can and should be studied using natural science methods. From this standpoint, behavior is shaped by functional relationships with the environment, especially through operant conditioning processes, where the consequences of an action influence its future probability.^(58,59)^ The term "radical" reflects a commitment to viewing the organism as a whole, continuously interacting with its context, rather than parsing behavior into separate internal and external domains.^(60)^ The term also emphasizes a unified explanatory system grounded in environmental interactions and functional relationships.^(49)^ Due to the "radical" term and viewpoint, functional contextualism is also involved with the science of behavior analysis.

Functional contextualism is a modern philosophical foundation for behavioral analysis that extends and complements radical behaviorism by emphasizing the prediction and influence of behaviors with precision, scope, and depth, always in relation to the context in which they occur.^(61,62)^ It is rooted in pragmatic philosophy, particularly in the work of William James and John Dewey, and aligns with the naturalistic and anti-dualistic stances of radical behaviorism.^(63-65)^ What distinguishes functional contextualism is its focus on the act-in-context as the unit of analysis, which considers behavior as inseparable from the historical and situational environment in which it is embedded.^(65,66)^ As such, functional contextualism provides the epistemological basis for various contemporary behavioral approaches, maintaining coherence with the principles of radical behaviorism through a shared emphasis on environmental interaction and empirical pragmatism.^(67)^

This contextual orientation is especially relevant when analyzing psychological phenomena through the lens of the diathesis-stress model. Both diatheses and stressors can be interpreted as temporally extended variables; the former representing elements of an individual's reinforcement history and behavioral tendencies, and the latter referring to current and evolving contingencies that occasion, maintain, or exacerbate certain behavioral patterns. From this standpoint, behavior is always embedded in context; it is the product of an ongoing transaction between individuals and their environment and not the expression of internal defects isolated from historical and situational factors.

Conceptualizing diathesis from a behavior-analytic perspective requires moving beyond the notion of fixed traits or internal predispositions. Instead, we must recognize how learning histories, rule-governed behaviors, relational frames, and verbal communities shape what may appear to be "vulnerabilities." For instance, a "perfectionistic" trait may reflect a history of reinforcement of high standards and avoidance of failure, contextualized by social contingencies that punish ambiguity or perceived incompetence. Similarly, stressors are not merely external "pressures" but must be understood functionally—as conditions under which certain behavioral repertoires are more or less effective, more or less likely to contact reinforcement or punishment. Therefore, this model is monistic and functionalist^(49)^ and in line with radical behaviorism. Rather than positing a dualistic separation between mind and body, behavior is understood as a unified phenomenon resulting from the dynamic interaction between an organism and its environment.^(68)^ Thoughts, emotions, and other "mental" events are considered forms of behavior that are influenced by, and in turn influence, biological and environmental contingencies. While process philosophy and embodied cognition also resist reductionism, this model can be situated within a behavior-analytic tradition that emphasizes operant processes and the role of learning history in shaping functional repertoires, which is one of the goals of this paper.

From this perspective, intervention becomes a matter of altering the contingencies under which behavior occurs rather than correcting faulty brain chemistry. This may involve changing the discriminative stimuli, consequences, relational networks, and functional alternatives available to the individual, through methods such as behavioral therapy, environmental redesign, social skills training, community interventions, or culturally sensitive psychoeducation.

Philosophically, this approach embraces a monistic, functionalist view, in which all psychological phenomena, including thoughts, emotions, and symptoms, are viewed as behaviors subject to the principles of selection by consequences. The diathesis-stress model, when interpreted through this lens, does not reject biological facts but reframes them as part of a larger behavioral system, where genes, neurotransmitters, thoughts, language, and social interactions all participate in the ongoing regulation of behavior. In other words, this model accommodates the integration of biological, behavioral, and contextual data within a unified ontological framework. Rather than seeking causal primacy at any single analysis level, the model promotes a layered understanding of psychological functioning. Genetic, neurochemical, historical, and situational variables are viewed as interdependent-not hierarchically ordered—and their interactions constitute the functional units of analysis. This stance permits scientific pluralism, while avoiding the pitfalls of either biological reductionism or unstructured eclecticism.

This integrative and contextual understanding has far-reaching implications for clinical practice, public health policies, education, and legal systems. It encourages a move toward flexible, inclusive systems of care that are sensitive to the multiplicity of factors influencing mental well-being and challenges the medicalization of distress, while calling for ethical responsibility in shaping environments that promote psychological resilience, adaptive learning histories, and equitable access to reinforcement across diverse populations. In summary, the diathesis-stress model, when aligned with radical behaviorist epistemology, offers a philosophically coherent and scientifically rich framework for understanding mental health. It resists the simplistic attributions of pathology, honors the complexity of human behavior, and orients clinical and social interventions toward being functional and context sensitive.

## CONCLUSION

Although the diathesis-stress model offers a compelling alternative to reductionist perspectives, it is crucial to acknowledge the enduring value of the biological model in enhancing our understanding of mental health. Neuroscience and pharmacology have provided essential insights into the mechanisms underlying psychological functioning, and biological interventions have improved the lives of numerous individuals with psychiatric disorders. The contributions of the biological model, particularly in cases of severe mental illness, remain foundational for effective care and must be integrated, rather than excluded, within a contextual understanding of behavior.

However, no single model can fully capture the complexity of human behavior. Mental health is inherently multidimensional, and the integration of biological and contextual approaches represents a more comprehensive and humane framework. Collaboration between medical and psychological professionals is desirable and indispensable to address the nuanced demands of mental health care. When psychiatrists, psychologists, behavior analysts, social workers, and other professionals work together, guided by models that respect both biological sensitivities and environmental influences, they are better positioned to deliver personalized, effective, and sustainable interventions. In this light, the future of mental healthcare lies in strengthening interdisciplinary dialogue and refining integrative models that bridge the gap between the brain and behavior, molecules and meaning, structure and function. The diathesis-stress model, understood as part of this broader synthesis, is not merely a theoretical framework, but a dynamic tool for collaborative, contextually informed, and functionally guided intervention.

Future guidelines for the use of the diathesis-stress model should emphasize the importance of interdisciplinary training and education to ensure that mental health professionals are adequately prepared to integrate biological, psychological, and contextual variables into assessment and intervention planning. This training must move beyond traditional silos of knowledge and foster a shared conceptual language among psychiatrists, psychologists, social workers, behavior analysts, and other professionals. The goal is to cultivate practitioners who are not only specialists in their respective domains but also capable of working collaboratively within a biopsychosocial framework, appreciating the unique contributions of each discipline.

Mental health systems should transition from categorizing individuals based on symptom clusters to a more functional and dynamic approach that considers how vulnerabilities and environmental stressors interact over time and context. This functional perspective enables more nuanced and personalized assessments, allowing clinicians to identify specific environmental contingencies, relational patterns, and behavioral repertoires that maintain or mitigate psychological distress. This reorientation would enhance diagnostic validity and clinical utility by prioritizing the functionality of behavior over rigid diagnostic labels.

Furthermore, future applications of the model should place stronger emphasis on prevention. Early intervention, before symptoms reach a diagnostic threshold, requires a systemic focus on reducing chronic stressors such as poverty, discrimination, social isolation, and unstable housing. Preventive interventions could include school-based social-emotional learning programs, community resilience initiatives, family-based support services, and workplace wellness policies. Mental health professionals can help reduce the incidence and severity of psychological disorders by proactively identifying and addressing environmental risk factors.

Ethical and cultural sensitivity must remain central to clinical decision-making. Diatheses and stressors are deeply influenced by an individual's cultural norms, belief systems, access to resources, and historical context. Behaviors that are considered maladaptive in one sociocultural context may be functional and adaptive in another. Therefore, practitioners must engage in culturally informed formulations and avoid pathologizing them without a deep understanding of their contextual relevance. This requires training in cultural humility and the use of assessment tools that reflect diverse values and lived experiences. Future research should increasingly focus on elucidating the interplay between biological factors, such as genetic predispositions, neurochemical profiles, and neurophysiological responses, and environmental contingencies, including reinforcement schedules, exposure to trauma, and social reinforcement networks. Integrative investigations can inform the development of multimodal interventions that combine psychopharmacological support with behavioral, cognitive, and systemic strategies. Evidence-based protocols that flexibly adapt to each individual's diathesis-stress configuration hold promise for more effective, personalized, and sustainable mental healthcare.

By advancing these research directions, the diathesis-stress model can continue to evolve as the cornerstone of a comprehensive and humane mental health paradigm. Its strength lies in its capacity to embrace the complexity of human behavior without descending into conceptual chaos, offering a structured yet flexible framework for understanding the interactive influence of biology and the environment on mental functioning. This perspective affirms the necessity of personalized care and the ethical imperative to consider the full spectrum of factors that define the human experience.

## Data Availability

The underlying content is contained within the manuscript.
